# Instructional Set Moderates the Effect of GRE on Faculty Appraisals of Applicant Competence: A Vignette Study With Implications for Holistic Review

**DOI:** 10.3389/fpsyg.2021.749621

**Published:** 2021-10-15

**Authors:** Isabelle Rios Hernández-Colón, Annmarie Caño, Lee H. Wurm, Gavin Sanders, Jennifer Nava

**Affiliations:** ^1^College of Education and Human Development, University of Maine, Orono, ME, United States; ^2^College of Arts and Sciences and Department of Psychology, Gonzaga University, Spokane, WA, United States; ^3^Department of Psychology, University of Detroit Mercy, Detroit, MI, United States

**Keywords:** standardized tests, GRE, graduate admissions, holistic review, competence, heuristics and biases

## Abstract

While there is movement to create more equitable and holistic admission review processes, faculty continue to place strong emphasis on a single piece of information when making admissions decisions: standardized test scores. This study used an experimental design to test whether instructions provided to faculty prior to assessing doctoral applicants could support holistic review by reducing the weight of the general record examination (GRE) in faculty appraisals of competence and merit for graduate study. Tenured and/or tenure-track faculty (*N*=271) were randomly assigned to one of three instructional conditions: Control (no instruction), “Diamond in the Rough,” and “Weed Out.” In addition, faculty participants were randomly assigned to read one of two vignettes of a prospective first-generation student who either received high or average GRE scores. Faculty then rated the applicant’s competence using a three-item survey. As expected, faculty who read the vignette describing the candidate with the high GRE rated him as more competent than faculty who read the average GRE vignette. In addition, being instructed to seek out diamonds in the rough buffered the effect of the GRE score on competence. Faculty were also asked to indicate whether they would need additional information to make an admissions decision. They were more likely to ask about grades and research skills than about psychosocial factors that might contextualize the candidate’s performance and perceived competence. The results of this study have implications for creating more equitable doctoral admissions processes that center equity, diversity, and inclusion in decision making.

## Introduction

As gatekeepers, faculty decide who merits access not only to graduate programs but also to careers that require advanced degrees, including the professoriate. The Council of Graduate Schools (Kent and McCarthy, 2016) and funding agencies, such as the National Science Foundation, have recognized that a key to solving today’s scientific and societal problems is to create more inclusive and equitable processes to diversify the workforce through holistic review processes. Holistic review includes a variety of practices aimed at contextualizing applicants’ academic and professional experiences rather than focusing on single data points, such as standardized test scores. Yet, research has demonstrated that standardized tests, such as the general record examination (GRE), are heavily weighted in admissions decisions. Sometimes, these scores are used to make initial selections of candidates deserving of further review (Miller and Stassun, 2014; Posselt, 2014, 2016). Yet, other research has shown that test performance is highly correlated with race, gender, and first-generation college student status (Educational Testing Service, 2019) and that an overreliance on these scores may bar access to graduate school for deserving students from diverse backgrounds (Smith and Garrison, 2005; Vasquez and Jones, 2006; Miller and Stassun, 2014; Gómez et al., 2021). A purpose of the current study was to explore whether simple instructions could be used to mitigate the outsized influence that GRE scores continue to have on faculty’s judgments of applicants for graduate study.

The current study extends the work of Cano et al. (2018), who conducted an experimental vignette study of faculty at a single institution. In a 2×2 design, faculty were randomly assigned to read a vignette about a doctoral applicant in which GRE scores and first-generation college student status were manipulated. Whereas faculty who reviewed the higher GRE vignettes were more likely to interview the candidate, faculty members’ empathic orientation moderated this effect. Specifically, in the average GRE group, greater empathy in faculty was associated with a higher likelihood of interviewing, with interview rates appearing to be equivalent to vignettes with higher GRE scores. Faculty who were themselves first-generation college students were more likely to admit the applicant with average GRE scores and whose vignette included mention of their first-generation college student status. These findings are consistent with the empathy literature, which shows that empathy and shared life experience can influence altruistic and prosocial behavior toward others (Davis, 1980; Batson et al., 2007). Taking the perspective of applicants and empathizing with them may lead faculty to evaluate the experiences of marginalized candidates in a more favorable or generous light.

### Are Faculty Judgments Malleable?

The research described above is one of many examples of how decision making is a process that is subject to our personal experiences. Decision making is also subject to biases and heuristics (Tversky and Kahneman, 1974). Faculty may use heuristics or cognitive shortcuts to simplify admissions decision making because of the need to review a great deal of information in a limited period of time. To make more efficient decisions, faculty may rely on their own personal experiences serving on search committees (e.g., availability heuristic), memories of successful or unsuccessful students (e.g., representative heuristic), and traditions, stories, and assumptions in their disciplines regarding adequate preparation for graduate study. At the same time, relying on these heuristics may replicate long-standing assumptions that deny educational opportunities to qualified candidates. It is possible to intervene and short-circuit these heuristics by encouraging deeper information processing (Kahneman, 2011). And indeed, there is growing interest in looking into ways to change how faculty members make admissions decisions.

In her study of the working of doctoral admissions committees, Posselt (2014, 2016) found that faculty members receive little guidance during the process. They often use unwritten norms and personal experience in selecting candidates, which often recreates or perpetuates patterns of admissions that favor continuing generation graduates from elite institutions and who received high test scores, which limits diversity in the graduate student pool. Posselt and others have called for department heads and graduate directors to reimagine doctoral admissions by creating rubrics that specify experiences and qualities that are valued by the program. Indeed, many programs have adopted holistic admissions and other methods that provide direction to faculty members (Mathur et al., 2019).

In the current study, we experimented with simple prompts that make explicit some of the ways in which faculty may approach the evaluation of doctoral applicants. We include a control condition that mimicked a “business as usual” approach to reviewing applications. Faculty members randomized to this condition were told to evaluate the candidate for admissions to their doctoral program. We also included two other conditions that primed faculty members to read the vignette of the doctoral applicant with particular goals. In one condition, faculty were instructed to look for the “Diamond in the Rough” candidate who could succeed in their program. In the other, faculty were instructed to look for the candidate that should be avoided because they will not succeed in their program (the “Weed Out” condition). The purpose of including these three instructional sets was to examine the extent to which instructional sets might mitigate the effect that GRE scores have on faculty members’ perceptions of competence.

### Current Study

The purpose of this study was to examine the effects of standardized test scores and instructional sets on faculty perceptions of the competence of a doctoral applicant. We focused on faculty members’ ratings of competence rather than their likelihood of interviewing or admission because participants in Cano et al. shared that they would rarely make admissions decisions based on the limited information provided in the vignette. Thus, assessing perceived competence is more ecologically valid.

The Cano et al. vignettes for first-generation college students were used, which described a male candidate’s skills and experiences along with his GPA and test scores. In both cases, the candidate had a first-year GPA that was less than a B and a final GPA that was approximately a B+. The only information that differed between the vignettes were GRE scores. This permitted a comparison of evaluations for high (75th percentile) and average (50th percentile) test performance. It is expected that faculty who were randomly assigned to the high GRE vignette would view the candidate as more competent.

Prior to reading the vignettes, faculty participants were randomly assigned to read one of three sets of instructions to test the extent to which the framing of the review process impacts judgments of competency. As noted above, the three conditions included no guidance (Control), seeking the Diamond in the Rough who can succeed, and Weeding Out the student who cannot succeed. It is expected that the instructional set would modify their ratings of competence based on whether the candidate had high or average GRE scores.

Finally, faculty members were asked if they needed additional information (e.g., specific grades and research skills) to interview or admit the hypothetical candidate. This item was included to provide insights into how participants’ contextualize students’ applications during their decision-making process. Along with instructional sets to committees, this information provides insights that can inform holistic review interventions.

## Materials and Methods

### Procedure

This study was approved by the Institutional Review Board at Wayne State University. Faculty members at six urban Carnegie classified “Highest Research Activity” doctoral universities across the United States were recruited to participate in this study. Publicly available email addresses were collected by searching the public Web sites of these universities for tenure-track/tenured faculty in the science, technology, engineering, and mathematics (STEM) and social, behavioral, and economic sciences (SBE) disciplines. Emails that included the purpose of the study and a link to the online Qualtrics survey were then sent three times over the course of 3weeks to potential participants. Potential participants were told that the purpose of this study was to better understand how faculty members make doctoral admissions decisions. Informed consent was obtained *via* an information sheet that opened upon clicking the survey link.

A total 2,756 faculties were emailed and 344 initiated (i.e., clicked on the survey link to begin the survey) the survey. Of those who initiated the survey, 344 completed at least one item. For the purposes of this study, we only include participants who completed the study, which resulted in a sample size of *N*=271.

After reading the online information sheet, participants were randomly assigned to one of three instruction sets: Control, “Diamond in the Rough,” and “Weed Out.” In the Control condition, the instructions were as: “Your task is to evaluate applicants to your doctoral program. Please consider the information about the candidate that appears on the next page and then answer the questions that follow.” Diamond in the Rough participants were instructed as: “Your task is to find “Diamond in the Rough” applicants who can succeed in your doctoral program. Please consider the information about the candidate that appears on the next page and then answer the questions that follow.” Finally, Weed Out participants were told as: “Your task is to Weed Out applicants that will not succeed in your doctoral program. Please consider the information about the candidate that appears on the next page and then answer the questions that follow.”

After reading the instructions, participants read a vignette about a male first-generation college student candidate who was applying to a doctoral program:

Joe is an undergraduate in his senior year at a large public university and he has applied to your doctoral program. Joe indicated in his personal statement that he is pursuing graduate studies to prepare to be a professor and a researcher. Joe identified you as a potential advisor because he is interested in your program of study. It is clear from his personal statement that he has read several recent articles of yours and appears to understand the importance of the work presented in them.

To prepare himself for this career, Joe has taken the necessary prerequisite coursework for the doctoral program. In college, Joe volunteered as a research assistant for a faculty member for 1year. During this experience, he learned how to collect and enter data into Excel and SPSS, conducted descriptive analyses, and participated in weekly lab meetings with the professor, graduate students, and several other undergraduates. He noted that this experience was beneficial in helping him to recognize that he could pursue a career in scholarly research, especially given that he was the first in his family to attend college. Joe has also noted in his statement that he volunteered at a social service organization once per week. Joe wrote that his research and volunteer experienced helped him develop skills to work effectively on his own and in a team. Joe has also mentioned that he has learned good organizational and leadership skills by working a part-time job at a dining hall on campus during which he was able to work his way up the ranks from server to manager.

Respondents were randomly assigned to receive one of two sets of scores for Joe. Whereas both sets of scores included an overall GPA of 3.2/4.0 and a first-year GPA of 2.75, one group included higher GRE scores (GRE Verbal=75th percentile, GRE Quantitative =80th percentile, and GRE Analytical=60th percentile) than the other (GRE Verbal =55th percentile, GRE Quantitative=40th percentile, and GRE Analytical=50th percentile). These GRE ranges were selected based on two of the authors’ experiences as search committee members (AC and LW) as well as to be sufficiently different from each other but not so extremely high or low as to be unrealistic representations as to arouse suspicion from participants.

### Measures

After reading the instructions and vignette, participants were then asked to rate Joe’s competence for graduate study with a three-item scale developed by Moss-Racusin et al. (2012). Items included as: “Did the applicant strike you as competent?,” “How likely is it that the applicant has the necessary skills for this job?,” and “How qualified do you think the applicant is?” Participants responded using a 1 (not at all) to 7 (very much) scale. The inter-item reliability for competence rating was excellent (Cronbach’s alpha=0.94).

Participants were able to indicate if they wanted to review additional information about Joe to make admissions decisions: “What, if any, additional information would you like to know about Joe or his application to make a decision to *interview/admit* him?” Choices included as: No additional information needed, specific research skills, grades in courses, communication (oral and/or writing) skills, interpersonal skills, additional demographic information (e.g., race/ethnicity), volunteer or civic/community service or engagement, personal history or experiences including obstacles overcome, and other (fill in the text box).

Participants then responded to survey items to assess demographics (e.g., sex, degree year, and academic discipline).

## Results

### Descriptive Statistics

The mean age of participants was 50.71 (SD=12.39). Almost all of the participants had served on a graduate admissions committee (91.14%, *n*=247) and had earned a Ph.D. (98.52%, *n*=267). Table 1 displays the other demographic information for the sample. Data were not available for all demographic characteristics as participants were permitted to skip items they did not want to disclose.

**Table 1:** Participants’ demographic characteristics.

Participant Demographics^*^	
Variable	% (n)
*Race/Ethnicity* ^**^	
White	85.24% (231)
African American/Black	2.58% (7)
Asian	8.49% (23)
Hispanic/Latina/o/x	7.83% (17)
First People/American Indian/Alaskan Native	0% (0)
Native Hawaiian or Pacific Islander	0.37% (1)
Other	1.85% (5)
*Biological Sex*	
Male	62.36% (169)
Female	36.16% (98)
Prefer Not to Say	1.48%(4)
*Discipline*	
STEM	58.67% (159)
SBE	39.48% (107)
Arts and Humanities	0.74% (2)
Other	1.11% (3)
*Faculty Track (Tenure-Track or Tenured)*	
Yes	97.79% (265)
No	1.48% (4)

However, frequencies may not sum to 271 for each variable as participants were permitted to skip individual questions.

* *N=* 271.

** Participants were able to choose all identities that apply.

### Interactions Between Instructional Set and Vignette

Data were analyzed using version 4.0.5 of the R statistical programming language (R Core Team, 2021), along with the car (version 3.0.10) and effects (version 4.2.0) packages by Fox and Weisberg (2019).

Mean competence scores were analyzed in a 2×3 factorial ANOVA, with vignette (high GRE vs. average GRE) and instructional set (Control, Diamond in the Rough, and Weed Out) as independent variables. This analysis was conducted to examine the extent to which vignette (high GRE vs. average GRE) and instructional set (Control, Diamond in the Rough, and Weed Out) interacted to predict faculty participants’ perceptions of the applicant. The results of this analysis are shown in Table 2.

**Table 2:** Results of the 2×3 factorial ANOVA for mean competence scores.

Source	SS	df	MS	F	p	effect size eta^2^
Vignette	177	1	177.000	12.837	0.0004	0.046
Instructional set	29	2	14.500	1.040	0.3550	0.008
Vignette x instructional set	113	2	56.500	4.103	0.0176	0.030
Error	3,659	265	13.808			

There was a significant main effect of vignette, demonstrating that participants rated Joe as more competent and qualified if they were assigned the high GRE vignette. In addition, there was a significant vignette x instructional set interaction.

**Figure 1:**
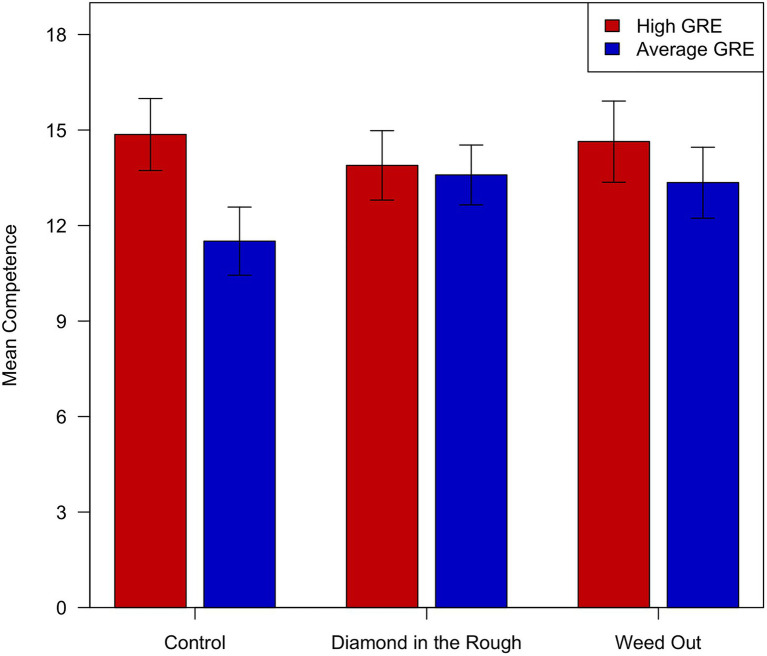
Mean competence scores as a function of vignette and instructional set. Error bars show 95% confidence intervals.

**Figure 2:**
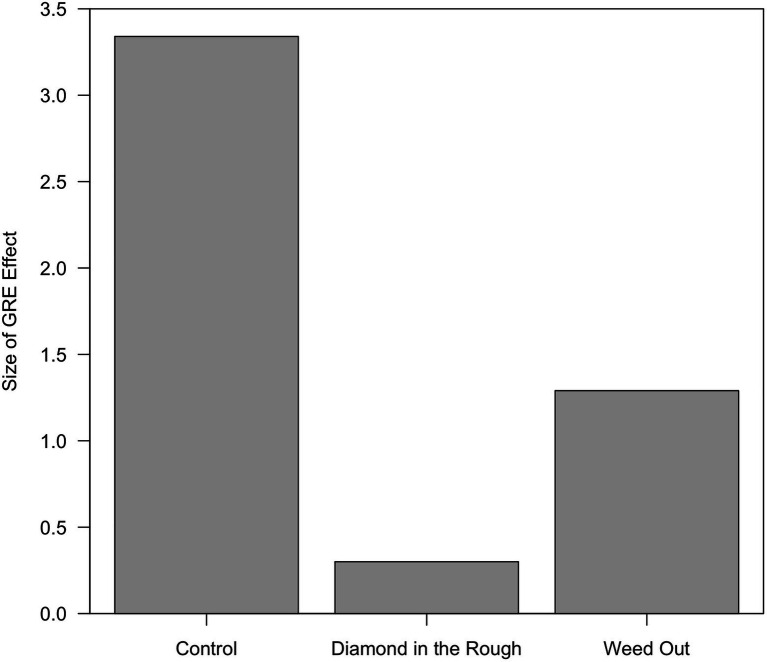
The difference between the mean competence scores in the high GRE and average GRE vignettes as a function of instructional set.

As shown in Figure 1, the difference between the mean competence scores was greatest in the Control condition, was negligible in the Diamond in the Rough condition, and was intermediate in the Weed Out condition. These by-condition GRE effects are shown in Figure 2. They were explored further by means of *t*-tests.

In the Control condition, the mean competence score for the high GRE vignette was 3.34 greater than the mean competence score for the average GRE vignette. This difference was significant [t(87)=3.807, *p*=0.0003]. The analogous differences in the other two conditions were smaller, and neither was significant [for Diamond in the Rough, t(104)=0.433, *p*=0.666; for Weed Out, t(74)=1.605, *p*=0.113].

### Additional Information Requested by Faculty

Participants were able to indicate if they wanted to review other information about the applicant. Table 3 shows the frequency with which faculty participants desired additional information before making a decision to interview or admit the candidate into their graduate program. Faculty participants endorsed different types of information in similar proportions whether they were making an interview or admissions decision. Specifically, common pieces of information include grades in particular courses relevant to the field of study, more specificity about research skills, and examples of communication skills. Note, however, that the least requested information tended to be psychosocial information (i.e., demographic information, interpersonal skills, and community engagement) that could be used to contextualize an applicant’s portfolio, including obstacles or challenges the student overcame or strengths that may enhance graduate student success.

**Table 3:** Desired information before making an admissions-related decision.

	Type of Admissions Decision	
Type of Information Desired	Interview% (n)	Admit% (n)
Grades in courses	19.3% (73)	16.1% (61)
Specific research skills	14.2% (54)	13.2% (50)
Communication (oral and/or written) skills	13.2% (50)	14.2% (54)
No additional information needed to make an admissions-related decision	10.3%(28)	2.6% (7)
Personal history or experiences including obstacles overcome	5.8% (22)	8.4% (22)
Interpersonal skills	5.5% (21)	5.5% (21)
Additional demographic information (e.g., race and class)	3.7% (14)	3.2% (12)
Volunteer, civic, community service, or engagement	0.5% (2)	0.8% (3)

## Discussion

Based on the need to identify pragmatic solutions to support holistic review, the purpose of this study was to examine the extent to which instructional sets could affect faculty members’ judgments of competence about graduate program applicants. More specifically, this study tested whether different types of instructions could modify the strong effect that standardized test scores often have in graduate admissions decision making. As expected and in line with research showing the weight that the standardized test scores have on judgments of merit (Croizet, 2008; Posselt, 2014, 2016), faculty who were randomly assigned to read the vignette with the high GRE rated the applicant as more competent and qualified than faculty who were assigned to the average GRE vignette. Recall that there were no other differences in the vignettes than the GRE scores. The results mean that, all things being equal, faculty use standardized test scores to make appraisals of competence. It is somewhat disturbing to see that one piece of data continues to outweigh so significantly other evidence, especially when the Educational Testing Service (2019) has argued that decisions should not be made on this single piece of evidence. At the same time, this result was not surprising given that people take mental shortcuts to make decisions in a more efficient manner (Tversky and Kahneman, 1974). Faculty have personal and collective professional experiences that may make them more susceptible to heuristics like the availability and representative heuristics when considering information like the standardized test scores.

Yet the current findings show that the outsized role of the GRE effect is not inevitable, which may be heartening for faculty and staff who are attempting to build holistic review processes. Faculty in the current study also provided different competency ratings to the applicant depending on the instructions they received. Specifically, faculty receiving the “Diamond in the Rough” instructions rated the candidate’s competency similarly regardless of his GRE score. While not significantly different, high and average GRE candidate competence ratings were somewhat more disparate in the “Weed Out” condition. The largest difference was between the mean competence scores for faculty receiving no instruction (Control condition). On average, faculty receiving no instruction provided a competence rating that was more than 3 points higher for the high versus the average GRE candidate. The Control condition most closely approximates “business as usual” in graduate programs, where faculty are provided portfolios to review with no instruction as to how to review them. If this is the case, the typical approach to reviewing graduate applicants results in decisions in which one piece of information carries the weight in review.

Returning to the two experimental conditions, faculty who read the “Diamond in the Rough” instruction provided similar ratings of competency regardless of GRE score. Perhaps faculty who read this prompt reviewed the vignette more closely and noticed that the candidate was able to improve their GPA over time and had taken the initiative to get research experience, diminishing the weight of the GRE in their appraisals of competence. It is interesting that the GRE had little effect on competency scores when faculty were presented with the “Weed Out” instruction, although the difference fell in between the “Diamond in the Rough” and control conditions. Perhaps providing any instruction, even if it is to select the “worthy few,” charges faculty with more deeply processing the information provided in the vignette. That is, paying attention to the details of a candidate’s portfolio may reduce the impact of a single piece of data that might ordinarily carry great weight in snap decision making.

To further understand how faculty use information to make admissions decisions, we also asked participants to indicate what additional information they would need to extend an invitation to interview and to admit the candidate. When they indicated they wanted to see additional information, faculty participants were most likely to request academic information, such as grades in specific courses relevant to the field of study, specific research skills, and examples of communication skills. Interestingly, psychosocial information, which could be used to further contextualize an applicant’s experience, was requested less frequently: obstacles or challenges the student overcame, demographic information including race and gender, interpersonal skills, and community engagement activities. Information in these areas could be used to explain the candidate’s low initial GPA and increases in GPA over time, especially as the candidate was a first-generation college student. In addition, this type of information could provide valuable information about the candidate’s strengths in navigating environments characterized by systemic racism and working for justice in their communities. The fact that faculty asked for this information less frequently suggests that faculty may benefit from more guidance regarding how to contextualize applications and reduce implicit (or unconscious) biases that have been acted upon toward applicants from marginalized groups (Corrice, 2009; Milkman et al., 2015; Moss-Racusin et al., 2012).

The current findings must be interpreted in light of the study’s limitations. The fact that faculty were not compensated for their time to complete the study may have contributed to our low response rate. Our response rate may also be a function of recruiting a bulk of participants in spring and summer. Nevertheless, the study includes faculty from a number of institutions. Researchers wishing to continue this work can build upon these findings by offering compensation and conducting focus group interviews or open-ended survey questions to gather more information about how faculty appraise applicant competence and attempt to make admissions decisions, especially in the context of holistic review. In addition, researchers are encouraged to examine how faculty-staff decision making across the academic training pathway (e.g., K-12 education and access to academic camps and enrichment, college admissions, college course, and lab experiences) results in many opportunities to grant access (or not) to qualified students even before they reach the doctoral admissions stage.

The current study demonstrates that although standardized test scores continue to dominate in appraisals of graduate applicant merit, simple instructional sets can diminish the outsized effect of standardized test scores in judging applicants’ competence. In light of recent research demonstrating that the predictive validity of standardized tests is minimally meaningful and can hamper the goals of programs to create more just and diverse environments (e.g., Croizet, 2008; Pacheco et al., 2015; Petersen et al., 2018; Gómez et al., 2021), these findings have implications for the pursuit of Inclusive Excellence (Association of American Colleges and Universities, 2002). While a number of programs have eliminated a GRE requirement for doctoral admission (Langin, 2019), a number of programs still require or allow for optional submission of this information. For these programs, committees can consider the types of prompts they use to ensure their holistic admissions goals are met and they can be guided to request and evaluate information that can contextualize applicants’ experiences and skills to select competent students who will thrive in their programs and beyond.

## Data Availability Statement

The raw data supporting the conclusions of this article will be made available by the authors, without undue reservation.

## Ethics Statement

The studies involving human participants were reviewed and approved by the Wayne State University Institutional Review Board. Written informed consent for participation was not required for this study in accordance with the national legislation and the institutional requirements.

## Author Contributions

IH-C, GS, and JN contributed to data collection, writing, and analysis. AC and LW conceived the original idea for this study and contributed to writing and statistical analysis. All authors contributed to the article and approved the submitted version.

## Funding

This research was supported by the National Science Foundation: Michigan AGEP Alliance for Transformation (MAA) and Mentoring and Community Building to Accelerate Successful Progression into the Professoriate # 1305819. The corresponding author was a site PI.

## Conflict of Interest

The authors declare that the research was conducted in the absence of any commercial or financial relationships that could be construed as a potential conflict of interest.

## Publisher’s Note

All claims expressed in this article are solely those of the authors and do not necessarily represent those of their affiliated organizations, or those of the publisher, the editors and the reviewers. Any product that may be evaluated in this article, or claim that may be made by its manufacturer, is not guaranteed or endorsed by the publisher.
